# The COVID-19 pandemic in Brazil: space-time approach of cases, deaths, and vaccination coverage (February 2020 – April 2024)

**DOI:** 10.1186/s12879-024-09598-1

**Published:** 2024-07-18

**Authors:** Thaís Zamboni Berra, Yan Mathias Alves, Marcela Antunes Paschoal Popolin, Fernanda Bruzadelli Paulino da Costa, Reginaldo Bazon Vaz Tavares, Ariela Feh Tártaro, Heriederson Sávio Dias Moura, Letícia Perticarrara Ferezin, Monica Chiodi Toscano de Campos, Natacha Martins Ribeiro, Titilade Kehinde Ayandeyi Teibo, Rander Junior Rosa, Ricardo Alexandre Arcêncio

**Affiliations:** 1 University of São Paulo at Ribeirão Preto College of Nursing, Avenida dos Bandeirantes, Monte Alegre, Ribeirão Preto, São Paulo, 3900, 14040-902 Brazil; 2 Federal University of Tocantins. Palmas – Tocantins, Avenida NS-15, Quadra 109 - Alcno 14, Norte, s/n - bloco D - Plano Diretor Norte, Palmas, 77001-090 Tocantins Brazil

**Keywords:** COVID-19, Time Series Analysis, Spatial analysis, Real World evidence

## Abstract

**Objective:**

To assess the evolution of the COVID-19 pandemic in Brazil and its macro-regions, considering disease incidence and mortality rates, as well as identifying territories with still rising disease indices and evaluating vaccine coverage and population adherence to COVID-19 immunization.

**Methods:**

An ecological study conducted in Brazil with COVID-19 cases and deaths reported between February 2020 and April 2024, obtained through the Coronavirus Panel. Historical series were constructed from incidence and mortality rates to assess the pandemic’s evolution, and temporal trends were estimated using the Seasonal Trend Decomposition using Loess (STL) method. The Spatial Variation in Temporal Trends (SVTT) technique was employed to identify clusters with significant variations in temporal trends. Vaccination was analyzed considering the percentage of vaccinated and unvaccinated population in each municipality of the country.

**Results:**

Brazil recorded a total of 38,795,966 cases and 712,038 deaths from COVID-19 during the study period. Incidence and mortality rates showed three waves of the disease, with a fourth wave of smaller amplitude. Four clusters with significant case growth and two with increased deaths were identified. Vaccine coverage varied among municipalities, with some regions showing low vaccination rates and others with high immunization adherence.

**Conclusion:**

The study provided a comprehensive overview of coronavirus behavior in Brazil, and its results highlight the ongoing importance of vaccination and the need to direct efforts and resources to areas of higher risk.

**Supplementary Information:**

The online version contains supplementary material available at 10.1186/s12879-024-09598-1.

## Background

Since the emergence of SARS-CoV-2 in 2019 in Wuhan, China, COVID-19 has spread globally, causing millions of cases and deaths. By May 2024, Brazil had reported 38.7 million cases and 712.0 thousand deaths [1]. In this context, Brazil rapidly emerged as the second country in the Americas with the highest number of COVID-19 cases. Faced with alarming rates, scientists and authorities engaged in developing the first coronavirus vaccine, resulting in the development and testing of over 150 vaccines worldwide [2].

On May 5, 2023, the World Health Organization (WHO) announced the end of the Public Health Emergency of International Concern (PHEIC) related to COVID-19, after just over a year since the start of mass vaccination. This decision was made due to evidence of a decline in the numbers of deaths and hospitalizations associated with the disease, as well as considerable progress in immunizing the population against SARS-CoV-2. However, the WHO emphasized the importance of remaining vigilant as the virus is still circulating and can cause severe or fatal illnesses, especially in unvaccinated individuals or those with pre-existing health conditions [3].

Given the importance of understanding and monitoring the disease’s behavior, as well as identifying areas where the virus still poses a challenge, the analysis of epidemiological data becomes crucial. In light of the above, the objective of the present study was to assess the evolution of the COVID-19 pandemic in Brazil and its macro-regions, considering disease incidence and mortality rates, as well as identifying territories with still rising disease indices, and evaluating vaccine coverage and population adherence to COVID-19 immunization, between February 2020 to April 2024.

In this context, ecological studies play a significant role in identifying patterns and trends, contributing to decision-making and the development of strategies to combat the spread of the virus and minimize its impact on public health. The information obtained aims to provide relevant data to guide public health policies and pandemic control strategies, seeking to reduce virus transmission, minimize the impact on public health, and maximize the effectiveness of vaccination campaigns.

## Methods

### Study design and research scenario

An ecological study conducted in Brazil, a country of continental dimensions located in South America, with its capital in Brasília. Brazil presents enormous landscape, economic, and cultural diversity, being the largest country in South America, with approximately 203 million people and an area of over 8.5 million km².

The ecological analysis in the study used the divisions of the country. Brazil is subdivided into five macro-regions (north, northeast, south, southeast, and midwest), 26 federative units, in addition to the Federal District, composed of 5,570 municipalities.

### Study population and information sources

The population consisted of the total number of COVID-19 cases and deaths reported between February 2020 (when the first case was confirmed in the country) and April 2024, obtained through the Coronavirus Panel [4], provided by the Ministry of Health. We emphasize that, according to the Ministry of Health, the notification of COVID-19 cases is only done upon testing for the disease, and notification without diagnostic confirmation is not possible [5].

Data regarding COVID-19 vaccination were also considered, including the doses of the vaccine administered between January 2021 (the beginning of vaccination in the country) and April 2024, obtained through the Ministry of Health [6]. And the data regarding the resident population per municipality used to calculate rates (incidence, mortality and vaccination) were obtained through the new 2022 Census carried out by IBGE [7]. It is important to mention that all databases used to conduct this study were accessed and downloaded from the respective locations mentioned on May 8, 2024.

### Definition of cases and deaths by COVID-19

According to the Ministry of Health [8], given the non-specificity and heterogeneity of the clinical presentation of COVID-19 cases, a confirmed case is one with conclusive laboratory confirmation for SARS-CoV-2, regardless of signs or symptoms. However, in the absence of confirmatory diagnostic tests for all suspected cases, health services had to adopt a very sensitive suspected case definition, especially for mild symptomatic cases, considering the reported symptoms, due to the recommendation that people not seek hospitals and health centers for clinical assessment in the absence of respiratory distress as a sign of worsening disease [9].

Therefore, according to clinical-epidemiological criteria, a case is considered when there is a history of close or household contact with a laboratory-confirmed case of COVID-19, in the last seven days before the appearance of symptoms and when it was not possible to carry out laboratory investigation. Specific [8].

The same applies to deaths confirmed for COVID-19, which can also be [10]:

#### By clinical criteria

death due to SARS associated with anosmia (olfactory dysfunction) OR acute ageusia (gustatory dysfunction) with no other previous cause.

#### By clinical-epidemiological criteria

death from SARS with a history of close or household contact, in the 14 days prior to the appearance of signs and symptoms with a confirmed case of COVID-19.

#### By clinical-imaging criteria

death due to SARS that could not be confirmed by laboratory criteria and which presents at least one of the following tomographic changes.

- Peripheral, bilateral GROUND-GLASS OPACITY, with or without consolidation or visible intralobular lines (“paving”), OR.
- Multifocal GROUND-GLASS OPACITY of rounded morphology with or without consolidation or visible intralobular lines (“paving”), OR.
- REVERSE HALO SIGN or other findings of organizing pneumonia (observed later in the disease).

#### By laboratory criteria

death from SARS with test.

- MOLECULAR BIOLOGY: DETECTABLE result for SARS-CoV-2 carried out by the real-time RT-PCR method;
- IMMUNOLOGICAL: REAGENT result for IgM, IgA and/or IgG* carried out by the following methods: Enzyme-Linked Immunosorbent Assay - ELISA); Immunochromatography (quick test) for detection of antibodies; Electrochemiluminescence Immunoassay (ECLIA).
- ANTIGEN SEARCH: REAGENT result for SARS-CoV-2 using the Immunochromatography method for antigen detection.

Therefore, based on the definitions of the Ministry of Health, cases and deaths due to COVID-19 are considered to be those reported with ICD-10 [10], namely:

- B34.2 - Coronavirus infection of unspecified location (confirmed cases only).
- U07.1 - Diagnosis of COVID-19 confirmed by laboratory tests.
- U07.2 - Clinical or epidemiological diagnosis of COVID-19, when laboratory confirmation is inconclusive or not available.
- B97.2 - Coronavirus as the basic cause of diseases classified in other chapters of ICD-10.

### Vaccines available in Brazil and vaccination schedule

The race for an effective vaccine against the new coronavirus began almost immediately after the onset of the pandemic. Still in 2020 (first year of the pandemic) more than 150 vaccines were under development in the world and around six were in phase three, in which it is necessary to carry out clinical trials in different countries (and this is before the final phase, after from which comes – ideally – approval). At the end of August 2020, more than ten experimental vaccines were being tested on humans in Brazil [11].

From the beginning of vaccination, with vaccines approved under emergency authorization, to the present day, with vaccines being updated for new circulating strains of the coronavirus, in addition to including new age groups in the groups to be vaccinated. Currently, two vaccines that were used in Brazil have expired their emergency authorization, namely CoronaVac (Butantan Institute) and Bivalent Comirnaty B.A 1 (Pfizer) [12]. Furthermore, two other vaccines had their application suspended in Brazil, namely Sputnik V (Russia – Gamaleya Institute) and Covaxin (Bharat Biotech Limited International) [12].

The vaccines approved for use in Brazil and which are still available today are Comirnaty (Pfizer/Wyeth), Oxford/Covishield (Fiocruz and Astrazeneca), Janssen Vaccine (Janssen-Cilag), Covid-19 vaccine (recombinant) (Zalika) and Spikevax (Adium) [12]. In addition to these mentioned vaccines, two more vaccines received approval to be administered to the Brazilian population, but as a booster dose, that is, their recommendation is only valid for those individuals who have completed the primary vaccination schedule against COVID-19. These vaccines are called Bivalent Spikevax (Adium) and Bivalent Comirnaty BA.4/BA.5 (Pfizer) [12].

Further details about each of the immunizers mentioned here (including reason for suspension, technology used in development, date of registration of the immunizer, age range and recommended doses) can be found in Supplementary Material 1.

It is important to mention that according to the Brazilian Ministry of Health, the administration of two doses of the vaccine (or one dose in the case of single-dose vaccines) is considered the primary vaccination schedule against COVID-19 and the complete schedule as the administration of at at least two doses of the vaccine (or a single dose) plus a booster dose [13].

### Patient and public involvement

No patient involved.

### Statistical analysis

#### Time series and temporal trend analysis of cases and deaths by COVID-19

Initially, incidence and mortality rates for COVID-19 were calculated considering Brazil as well as each of its macro-regions. To calculate the rates, the monthly number of reported COVID-19 cases or deaths in the country (and macro-regions) (based on the notification date) was considered in the numerator, while the total population of the country (and macro-regions) was considered in the denominator, multiplied by the constant 100,000. It is important to mention that we consider the data released by IBGE in the last official census (2022) [7] as “population” for calculating monthly rates. Subsequently, monthly time series of the calculated rates were created for Brazil and its macro-regions, covering the period from February 2020 to April 2024.

Temporal series are characterized as a set of observations obtained sequentially over time and are typically analyzed based on their main components described as: trend, cycle, seasonality, and random variations. In the present study, the Seasonal Trend Decomposition using Loess (STL) method was utilized, which relies on locally weighted regression, to separate the components of the time series. Only the trend component was selected to graphically visualize the data behavior over time [14]. The analysis was conducted using RStudio software, employing the forecast package [15].

#### Spatial variation in temporal trends of cases and deaths by COVID-19

To detect areas with increasing trends for COVID-19 cases and deaths, the Spatial Variation in Temporal Trends (SVTT) [16] technique was employed, which aims to determine whether the temporal trend of this event is increasing or decreasing over time [17].

The temporal trends are calculated both within (Internal Temporal Trend - ITT) and outside (External Temporal Trend - ETT) the scanning circle. Therefore, the statistically significant aspect in this analysis is the temporal trends, not the formation of the cluster itself, as occurs in purely spatial and/or space-time scanning [16–20].

Brazilian municipalities were considered as the unit of analysis, so the geographic coordinates (latitude and longitude) of their centroids were obtained using ArcGIS software version 10.5. Additionally, the population considered was the annual population estimates obtained via IBGE and standardized by sex and age [7].

A discrete Poisson model was adopted, with no geographic overlap of clusters, clusters with circular shape, 999 replications in the Monte Carlo simulation, and the exposed population was stipulated at 3%, a value determined by the Gini coefficient, which is an effective tool for assessing the heterogeneity of cluster formation, revealing underlying patterns. Compared to the traditional approach, it identifies non-overlapping clusters more finely, considering situations where it is more sensible to report smaller, non-overlapping clusters rather than a single large cluster [18–20].

The analyses were conducted using SaTScan software version 9.3, and thematic maps were created using ArcGIS software version 10.5.

#### Spatial distribution of vaccination coverage

To evaluate the vaccination coverage and population adherence to COVID-19 immunization in Brazil, the vaccination rate for those who completed the vaccination schedule was initially calculated. A complete schedule was considered as the administration of at least two doses of the vaccine (or a single dose) plus a booster dose. Following guidelines from the Ministry of Health [13], the booster dose was only indicated for people over 12 years old, at first, when in 2023 some states expanded the age range. Therefore, for the present analysis, only people over 12 years of age were considered, minimizing possible views when considering the calculation involving children under 12 years of age.

The vaccination rate was calculated by considering the number of booster doses administered in the numerator and the total population over 12 years of age of the municipality in the denominator, multiplying the result by 100. By subtracting this value from the total of 100%, the percentage of the population that has not completed the COVID-19 vaccination schedule was obtained. It is important to mention that the population stratified by age group according to Brazilian municipalities was obtained through the last 2022 population census released by IBGE [7].

#### Spatial association analysis - incidence, mortality and vaccination

The incidence, mortality, and vaccination rates were subjected to the Getis-Ord Gi* technique to assess their spatial association in a univariate manner^17^. This technique is based on the Moran’s I index and is used to evaluate local spatial association using statistical distances, determining the degree of clustering for high and low values [21].

The Getis-Ord Gi* technique indicates local spatial association, considering the rates of each municipality based on a Queen-type neighborhood matrix, as in the present study. In this analysis, a z-score is also generated, where a higher z-score indicates more intense clustering of high values (Hotspot), while a lower z-score indicates more intense clustering of low values or a lower occurrence of the event (Coldspot) [21].

In addition to the z-score, the *p*-value and significance level (Gi-Bin) are provided, which identify statistically significant hot and cold spots. The values range from +/-3 and reflect statistical significance with a confidence level of 99%, +/-2 confidence level of 95%, +/-1 confidence level of 90%, and zero indicates no statistical significance [21].

For purposes of comparison with the completion rate of the vaccination schedule against COVID-19, the incidence and mortality rates were considered covering the period between 2021 and April 2024, when the immunizers became available. The maps related to the spatial association analysis and the percentage of people who have not completed the COVID-19 vaccination schedule were created using ArcGIS software version 10.5.

## Results

### Time series and temporal trend analysis of cases and deaths by COVID-19

Between February 2020 and April 2024, Brazil recorded a total of 38,795,966 cases and 712,038 deaths from COVID-19. Regarding reported cases, the highest numbers were observed in the Southeast, South, and Northt regions. As for the number of deaths, the Southeast region remained at the top of the list, followed by the South and North regions.

**Fig. 1:**
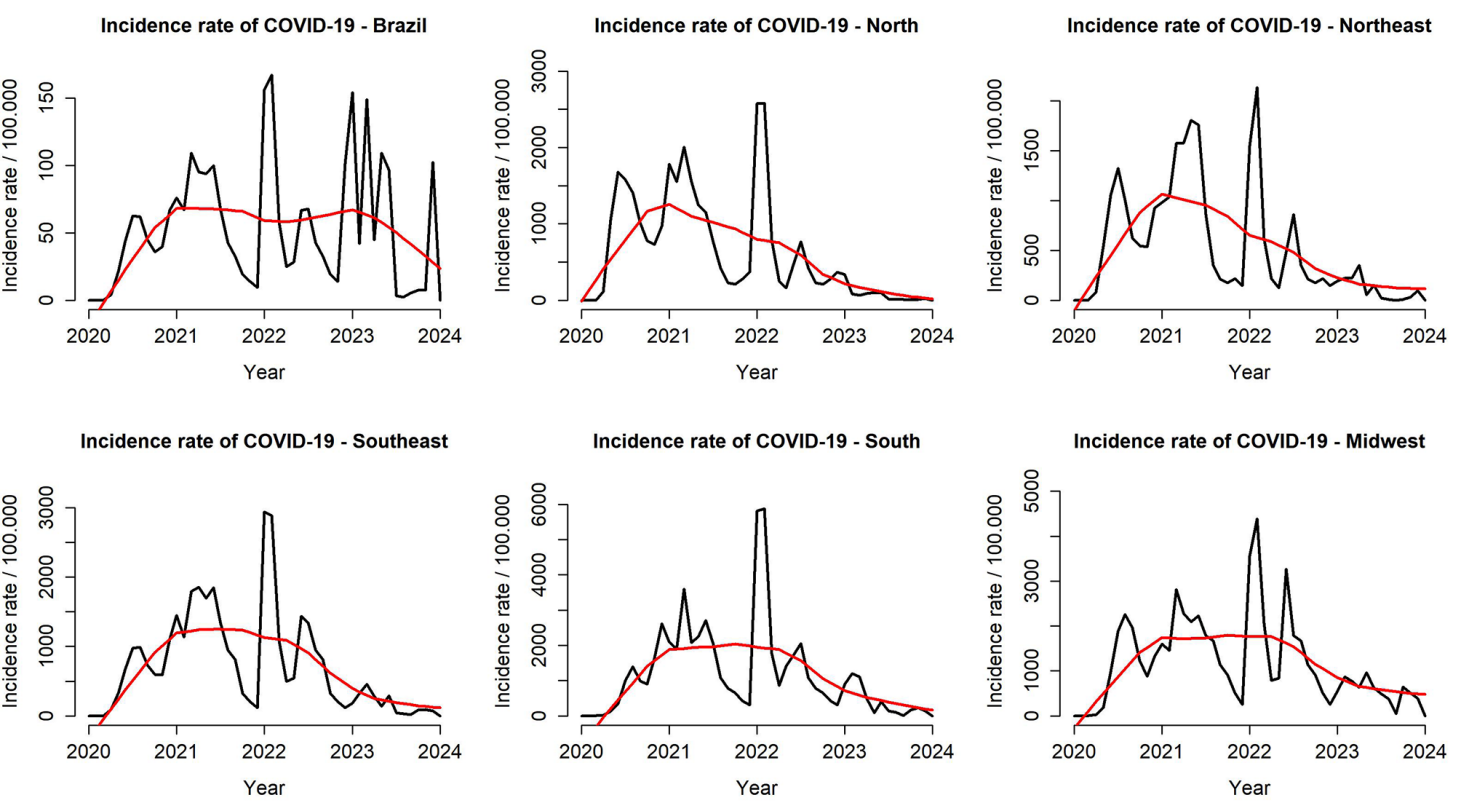
Monthly time series and temporal trend of COVID-19 incidence, Brazil (February 2020 - April 2024). Caption: **(A)** Monthly time series of COVID-19 incidence rate (black line) and its temporal trend (red line) in Brazil, estimated using the STL technique (February 2020 – April 2024); **(B)** North Region; **(C)** Northeast Region; **(D)** Southeast Region; **(E)** South Region; **(F)** Midwest Region

In Fig. 1, it is possible to observe that the incidence rates of COVID-19 show three main waves and a fourth smaller one. The first wave occurred in 2020, the second in 2021, and the third at the end of 2022 and the end of 2023. In mid-2022, there was a wave of smaller amplitude, except in the Central-West region, which recorded its second largest wave.

**Fig. 2:**
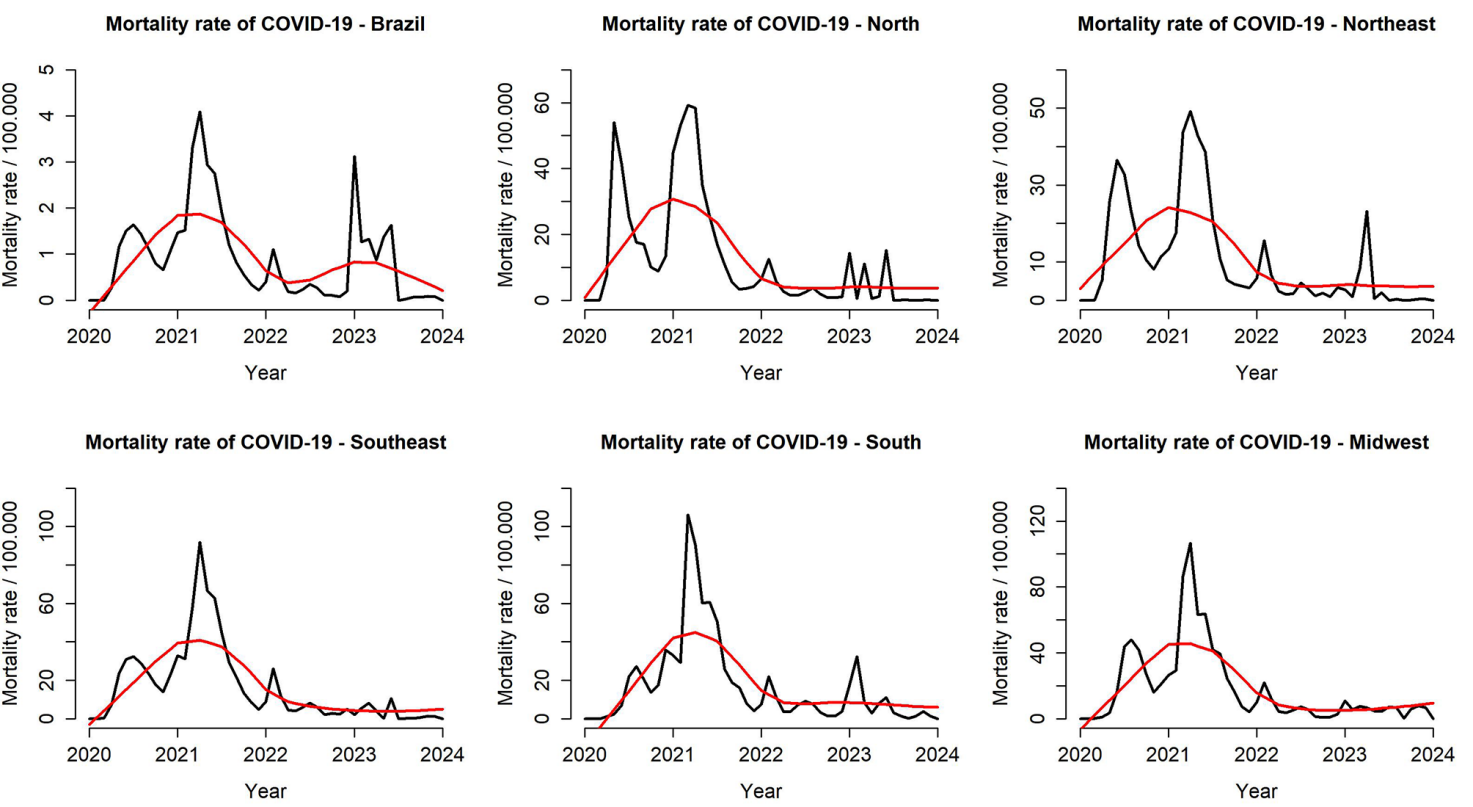
Monthly time series and temporal trend of COVID-19 mortality, Brazil (February 2020 - April 2024). Caption: **(A)** Monthly time series of COVID-19 mortality rate (black line) and its temporal trend (red line) in Brazil, estimated using the STL technique (February 2020 – April 2024); **(B)** North Region; **(C)** Northeast Region; **(D)** Southeast Region; **(E)** South Region; **(F)** Midwest Region

Figure 2 shows the COVID-19 mortality rates over time, revealing three main waves of deaths in the country and its macro-regions. The first occurred in 2020, the second spanned from the beginning to mid-2021, and the third, smaller one, was at the end of 2022 and the beginning of 2023.

### Spatial variation in temporal trends of cases and deaths by COVID-19

**Fig. 3:**
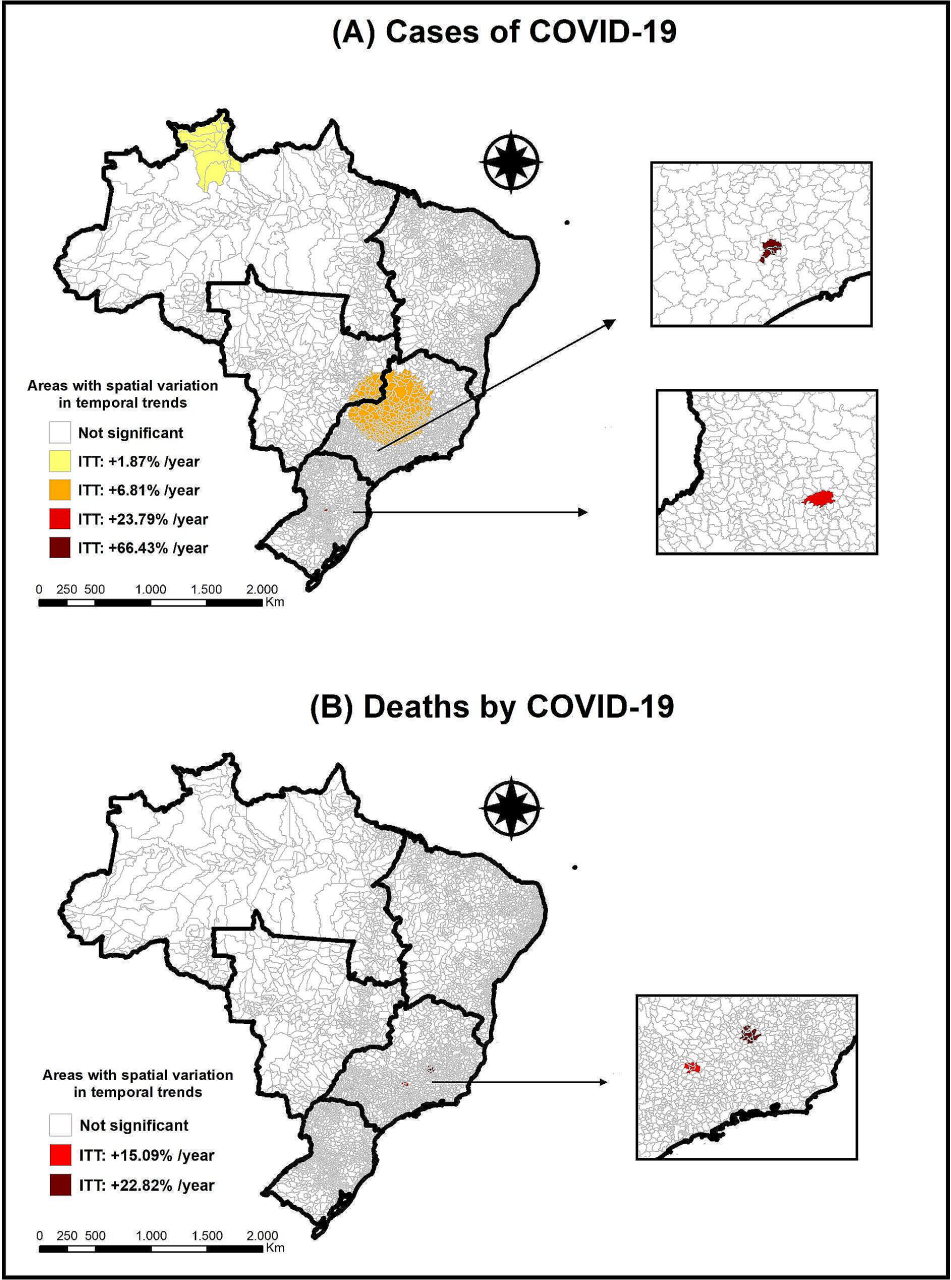
Areas with variation in the temporal trend for the occurrence of cases and deaths from COVID-19. Brazil (February 2020 - April 2024). Caption: **(A)** Areas with variation in temporal trend for the occurrence of COVID-19 cases in Brazil (2020–2024). **(B)** Areas with variation in temporal trend for the occurrence of COVID-19 deaths in Brazil (2020–2024)

Using the SVTT technique, an annual reduction of 14.68% in COVID-19 cases in Brazil was observed, aligned with the trend in Fig. 1. Four clusters were identified, indicating a significant increase (*p* < 0.01) with variations from + 1.87% to + 66.43% (Fig. 3-A).

Cluster 1 consisted of 15 municipalities in the North region, with a population of 450,479 inhabitants. There were 872,707 observed cases and 43,814 expected cases, resulting in an annual rate of 46,490.1 cases per 100,000 inhabitants. The ITT was + 1.87%, and the ETT was − 15.44% per year.

Cluster 2 comprised 463 municipalities in the Southeast and Midwest regions, with a population of 15,719,370 inhabitants. There were 2,632,285 observed cases and 1,528,884 expected cases, resulting in an annual rate of 4,018.5 cases per 100,000 inhabitants. The ITT was + 6.81%, and the ETT was − 17.86% per year.

Cluster 3 was composed only of the municipality of Concórdia, Santa Catarina, in the southern region of Brazil, with a population of 81,646 inhabitants. There were 86,026 observed cases and 6,674 expected cases, resulting in an annual rate of 30,084.3 cases per 100,000 inhabitants. The ITT was + 23.77%, and the ETT was − 14.83% per year.

Finally, Cluster 4 comprised 5 municipalities in São Paulo state (Southeast region), with a population of 701,672 inhabitants. There were 242,529 observed cases and 68,245 expected cases, resulting in an annual rate of 8,294.6 cases per 100,000 inhabitants. The ITT was + 66.43%, and the ETT was − 15.41% per year.

COVID-19 deaths showed a decreasing trend of 28.17% per year, also consistent with Fig. 2. Three clusters revealed significant increases (*p* < 0.01), with ITT ranging from + 15.09% to + 22.82% (Fig. 3-B).

Cluster 1 consisted of 5 municipalities in the Minas Gerais state (Southeast, region), with a population of 120,046 inhabitants. There were 929 observed deaths and 213 expected deaths, resulting in an annual rate of 185.7 deaths per 100,000 inhabitants. The ITT was + 15.09%, and the ETT was − 28.27% per year.

Finally, Cluster 2 was composed of 14 municipalities also located in the state of Minas Gerais, in the Southeast region of Brazil, with a population of 1,424,618 inhabitants. There were 10,115 observed deaths and 2,534 expected deaths, resulting in an annual rate of 170.4 deaths per 100,000 inhabitants. The ITT was + 22.82%, and the ETT was − 29.43% per year.

### Spatial distribution of vaccination coverage

**Fig. 4:**
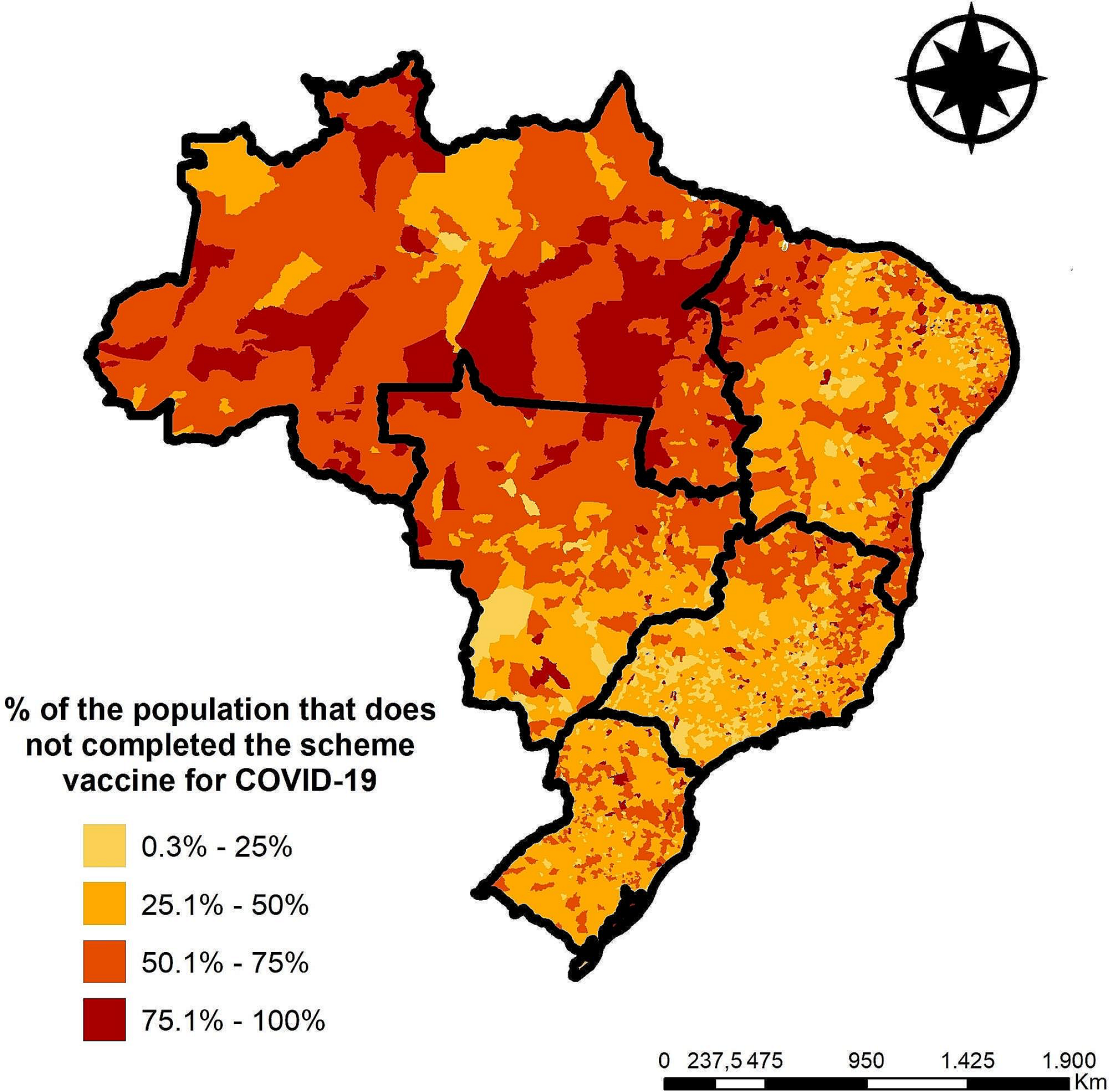
Spatial distribution of the percentage of people who have not completed the COVID-19 vaccination schedule. São Paulo, Brazil (February 2020 - April 2024)

Regarding COVID-19 vaccination, during the study period, 519,235,259 doses of available vaccines were administered to the Brazilian population, including 104,701,709 booster doses. Figure 4 presents the classification of Brazilian municipalities according to the percentage of people who have not completed the COVID-19 vaccination schedule. Of the 5,570 Brazilian municipalities, 44 municipalities (0.78%) achieved total immunization of their population, while 1,890 municipalities (33.93%) reported that more than half of their population had not completed the COVID-19 vaccination schedule.

### Spatial association analysis - incidence, mortality and vaccination

**Fig. 5:**
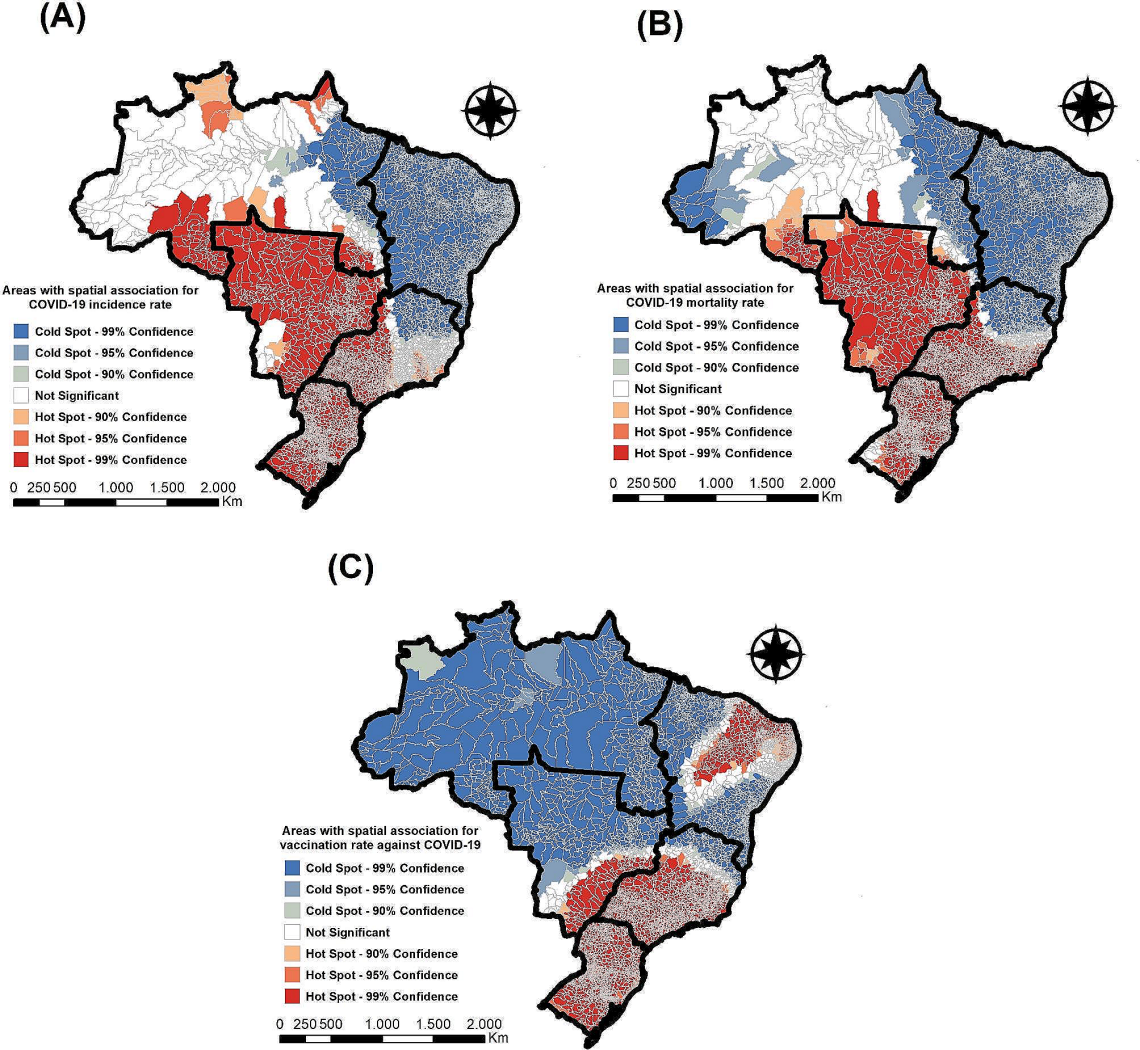
Areas with spatial association for COVID-19 incidence, mortality, and vaccination rates, Brazil (January 2021 - April 2024). Caption: **(A)** Areas with spatial association for the incidence rate of COVID-19 in Brazil (2021–2024). **(B)** Areas with spatial association for the mortality rate of COVID-19 in Brazil (2021–2024). **(C)** Areas with spatial association for the vaccination rate against COVID-19 in Brazil (2021–2024)

Finally, in Fig. 5A, it is possible to verify the areas identified with spatial association for the COVID-19 incidence rate. We identified 2,725 municipalities (48.92% of the country) with spatial association for high COVID-19 incidence rates, covering the South, Southeast, Central-West, and North regions of Brazil. Additionally, we identified 2,229 municipalities (40.01% of the country) with spatial association for low COVID-19 incidence rates, comprising the Southeast, Northeast and North regions of Brazil.

In Fig. 5B, which shows the areas identified with spatial association for the COVID-19 mortality rate, we observed a similar pattern to that found for the incidence rates, with 2,949 municipalities (52.94% of the country) identified with spatial association for high rates and 2,342 municipalities (42.04% of the country) with spatial association for low mortality rates from COVID-19.

Regarding COVID-19 vaccination rates (Fig. 5C), we identified 1,649 municipalities (29.60% of the country) with spatial association for low vaccination rates, covering the entire North region of the country, more than half of the Central-West region, and some municipalities in the Southeast and Northeast regions of Brazil. Additionally, we identified 3,410 municipalities (61.22% of the country) with spatial association for high vaccination rates, involving the entire South region of the country, more than half of the Southeast region, and some municipalities in the Central-West and Northeast regions of Brazil.

## Discussion

This study analyzed the evolution of the COVID-19 pandemic in Brazil from February 2020 to April 2024, considering incidence, mortality, and vaccination coverage. It identified the main waves of the disease and areas with significant trends.

The historical series revealed three major waves and a smaller fourth wave, highlighting the continued need for control, surveillance and vaccination. Beginning in 2020, the emergence of the new coronavirus presented the world population with a challenging scenario, with high rates of infection caused by the new disease, with large spikes in cases and deaths every year since its appearance, as shown in Figs. 1 and 2. The first waves of the disease, especially when we observe the deaths caused by COVID-19, were greater in scope, mainly due to the fact that it was a recent discovery, without studies or knowledge about its pattern of transmissibility, infectivity, lethality and risk factors for death [22, 23].

During the first year of the pandemic (2020) there were no vaccines and even today, there are no prophylactic or specific medications, or treatments proven to be effective specifically for this virus, with symptoms generally being treated [22, 23]. This fact, combined with the lack of scientific evidence and the abundance of contradictory information, generated confusion and uncertainty among the population and contributed to the rapid spread of the virus, which caused abrupt changes in social dynamics, resulting in high rates of morbidity and mortality [24].

Another factor that deserves to be mentioned and which also contributed to the increasing temporal trend in COVID-19 incidence and mortality rates observed until mid-2021 in Figs. 1 and 2, is the fact that the healthcare sector has also undergone significant transformations, with partial and restricted action, focusing mainly on coronavirus cases and which began to overload emergency rooms and hospitals. This led to the reallocation of professionals from other areas to COVID-19 units to meet the demand generated by the crisis [25].

Different regions of the country showed epidemiological behavior similar to the general pattern observed in Brazil as shown in Figs. 1 and 2, also corroborating the findings of the study by De Moraes et al. (2022) [22]. However, from the second half of 2021, this trend began to decrease. During this period, vaccines against COVID-19 were already available on the market and proven to be effective [26]. However, due to commercial and logistical issues, vaccination in Brazil started later compared to other countries in Europe and North America [27], which may have contributed to extending the time until rates declined, especially mortality.

The SVTT technique (Fig. 3) identified clusters with significant variations in case and mortality trends, indicating areas with acceleration or decline in the disease, requiring specific control and monitoring actions. Municipalities with high rates in the South, Southeast, Central-West, and North regions may receive priority attention for prevention and control. On the other hand, areas with low rates can serve as successful models to be replicated.

The Brazilian population is extensive and ethnically diverse, occupying various geographical regions. Unlike many European countries, the majority of the Brazilian population shows favorable acceptance of immunization against COVID-19, largely due to the country’s successful National Immunization Program (PNI) [28]. Even in a context where information and vaccine availability were not universal, vaccine acceptance in Brazil remains above 60% [28].

The analysis of vaccination coverage (Fig. 4) allowed us to identify municipalities with low vaccination rates, which is worrying, especially considering that some regions, such as the North region, have a significant proportion of municipalities in this category. The low vaccination rate may indicate logistical or communication challenges that need to be overcome to increase these rates. On the other hand, the identification of municipalities with high vaccination rates is encouraging and may reflect the success of immunization campaigns and population mobilization efforts.

A study [29] carried out in more than 23 countries identified misinformation as one of the main reasons for vaccine hesitancy in Brazil and around the world. The spread of fake news, particularly related to “adverse effects of vaccination”, increases vaccine hesitancy, as indicated in a study carried out in India [30]. Although it was identified that there was an increase in vaccine confidence in 2022 [29], hesitancy towards vaccination against COVID-19 remains substantial, corroborating the conclusions in Fig. 4.

We can see in Fig. 5 that some regions identified with spatial association for high vaccination rates coincide with locations identified with spatial association for high incidence and mortality rates. We know that the vaccine does not protect against coronavirus infection, but an opposite association was expected between areas of vaccination and mortality, that is, it was expected that places with a high spatial association for the vaccination rate would have a low spatial association for deaths. In some municipalities in the Northeast region of Brazil, this theory proved to be true, but in general it was not the pattern observed. A possible explanation for this could be related to the fact that with all the changes in social standards that the population has suffered due to this new threat, relationships of trust in the government and health services may have increased or decreased during the process of development of vaccines that is available today and with that, as disease rates increased, hope also increased for a vaccine that could reduce serious cases and deaths from COVID-19 and therefore, the population may have vaccinated more in these regions.

It is important to emphasize that all the results presented here are based on aggregated data and therefore do not provide individualized analysis of cases, deaths, and personal opinions regarding vaccination. However, they are crucial for guiding public policies and strategic health decisions, assisting in resource allocation, and implementing effective pandemic control measures. Furthermore, another limitation of the study is the use of secondary data, which may have led to unavailable or incomplete information, potentially affecting the accuracy of the results obtained.

Another important aspect to highlight is that this study considered only notified cases of COVID-19, and this notification is based on the diagnosis of COVID-19, which was performed only in symptomatic individuals tested for the disease. This may not fully represent the real epidemiological scenario of the state. It is also important to mention that the testing process for COVID-19 varied across the national territory and fluctuated over time. That is, there were periods of higher and lower testing, as well as locations that tested more than others, and this fact may cause a bias in temporal analyses, not reflecting the real epidemiological scenario at that time.

Finally, it is crucial that the results of this research be considered in conjunction with other epidemiological and demographic information, as well as with the continuous monitoring of the evolution of the pandemic. Therefore, it is essential for researchers, healthcare professionals, and managers at all levels to continue working together to address the challenges posed by this global disease and mitigate its impact on public health and society.

Furthermore, we encourage that new studies be carried out with a focus on understanding the dynamics of the disease specifically in municipalities identified with an increasing temporal trend for cases and deaths from COVID-19, in order to raise subsidies so that more accurate public policies can be designed to combat to new pandemics.

The results presented here should be interpreted and considered as part of an ever-evolving scenario. Continuous monitoring of the pandemic, along with updated research and analysis, is crucial to guide public policies, allocate resources, and implement effective measures to control COVID-19. Only through a collaborative approach across all levels can we overcome the challenges of the pandemic and ensure the safety and well-being of the entire population.

In conclusion, the study provided a comprehensive overview of coronavirus behavior in Brazil, and its results highlight the ongoing importance of vaccination and the need to direct efforts and resources to areas of higher risk.

### Electronic supplementary material

Below is the link to the electronic supplementary material.

Supplementary Material 1

## Data Availability

We declare that the data used in the present study are publicly accessible and without individual identification, which can be accessed through the Coronavirus Panel website, provided by the Ministry of Health (https://covid.saude.gov.br/). Data regarding COVID-19 vaccination were also considered, including the doses of the vaccine administered between January 2021 (the beginning of vaccination in the country) and April 2024 (https://infoms.saude.gov.br/extensions/SEIDIGI_DEMAS_Vacina_C19/SEIDIGI_DEMAS_Vacina_C19.html).

## Abbreviations

WHO: World Health Organization
PHEIC: Public Health Emergency of International Concern
STL: Seasonal Trend Decomposition using Loess
SVTT: Spatial Variation in Temporal Trends
ITT: Internal Temporal Trend
ETT: External Temporal Trend
Anvisa: National Health Surveillance Agency
PNI: National Immunization Program
