# Supplementary material for: The COVID-19 pandemic in Brazil: space-time approach of cases, deaths, and vaccination coverage (February 2020 – April 2024)
Source: BMC Infect Dis. 2024 Jul 18;24:704. doi: 10.1186/s12879-024-09598-1 (PMC11256571; doi:10.1186/s12879-024-09598-1)
Supplement: Supplementary file 1 — Supplementary Material 1 [file 12879_2024_9598_MOESM1_ESM.docx]

***Vaccines available in Brazil (February 2020 – April 2024)***

From the beginning of vaccination, with vaccines approved under emergency authorization, to the present day, with vaccines being updated for new circulating strains of the coronavirus, in addition to including new age groups in the groups to be vaccinated. Currently, two vaccines that were used in Brazil have expired their emergency authorization, namely:

**CORONAVAC (Butantan Institute)**

Anvisa approved its emergency use on January 17, 2021, which expired on May 21, 2023. On October 23, 2023, the company requested the closure of the vaccine registration process. While applied, two minimum doses were recommended, and for adults, the second dose should be applied between 2 and 4 weeks after the first. For children, the interval was 28 days. The authorized age range was for children aged 3 to 5, children and adolescents aged 6 to 17 and adults aged 18 and over. The vaccine was produced with inactivated virus antigen [1].

**Bivalent Comirnaty B.A 1 (Pfizer)**

Its registration approved on November 22, 2022, when Anvisa started to approve bivalent vaccines for booster doses against Covid-19. It was indicated as a booster dose and should be applied three months after the primary vaccine series or previous booster and indicated for populations over 12 years of age. The vaccine was produced with synthetic messenger RNA [2].

Furthermore, two other vaccines had their application suspended in Brazil, namely:

**Sputnik V (Russia – Gamaleya Institute)**

On April 1, 2021, Anvisa received a request to import the Sputnik V vaccine (Russia) and on June 6 of the same year, it released, under controlled conditions, part of the Sputnik import. Two doses of the vaccine are recommended, with the second dose being applied 21 days after the first and recommended for adults between 18 and 60 years of age. The vaccine is produced from Adenovirus D-26 D-5 [3].

It is important to mention that the Sputnik V vaccine should not be used in pregnant women, postpartum women, breastfeeding women, and individuals with comorbidities. It is also highlighted that the rare occurrence of severe cases of thrombosis syndrome with thrombocytopenia associated with the use of vaccines against COVID-19 that use adenovirus as a non-replicating viral vector has had a negative impact on the acceptability of vaccines from this platform, particularly when other vaccines have become available as an alternative [4]. At least one fatal case of this syndrome associated with the use of Sputnik V has been reported in the literature [5].

The import of the Sputnik V vaccine was authorized under Law 14,124/2021 and therefore the quality, safety and efficacy aspects of the vaccine were certified through the registration granted by the Russian health authority [3].

**Covaxin (Bharat Biotech Limited International)**

On March 23, 2021, Anvisa received a request to import the Covaxin vaccine and on July 24 of the same year, it closed the request for emergency use of the vaccine. Two doses of the vaccine were recommended, with the second dose being administered 28 days after the first; and was authorized in adults ≥ 18 years old and < 60 years old. The vaccine was produced from an inactivated virus and should also not be used in pregnant women, postpartum women, breastfeeding women, and individuals with comorbidities [6].

The suspension of imports of the vaccine was taken as a result of a statement from the Indian company Bharat Biotech Limited Intercional sent to Anvisa informing that the company Necessidade no longer has authorization to represent Bharat Biotech in Brazil [7]. In view of this, the import was authorized under Law 14,124/2021 and therefore the quality, safety and efficacy aspects of the vaccine were certified through the emergency use authorization granted by the Indian health authority [6].

The vaccines approved for use in Brazil, and which are still available today are:

**Comirnaty (Pfizer/Wyeth):** registration granted on 02/23/2021

The updated vaccine for the omicron XBB.1.5 strain, manufactured from synthetic messenger RNA and is authorized for application from 6 months of age, with a single dose for already vaccinated individuals and three doses for children up to 4 years of age who have not received any vaccine against COVID-19 [8].

**Oxford/Covishield (Fiocruz and Astrazeneca):** registration granted on 03/12/2021

A minimum of two doses is required for immunization, with the second dose being applied between 4 and 12 weeks after the first. The application of a booster dose (third dose) was approved, administered at least 6 months after completion of the primary vaccination schedule. Recommended for the population over 18 years of age and produced from a recombinant adenovirus vector. It is important to mention that in Communiqué GGMON 005/2021, it deals with the immediate suspension of the use of the AstraZeneca/Fiocruz Covid-19 vaccine in pregnant women due to complications [9].

**Janssen Vaccine (Janssen-Cilag):** registration granted on 5/04/2022

Single dose vaccine. The application of a booster dose (second dose) is authorized at least 2 months after the primary vaccination in individuals aged 18 years or over. Produced from adenovirus serotype 26 (Ad26) vectors [10].

**Bivalent Spikevax (Adium):** registration granted on 06/26/2023

The bivalent Spikevax vaccine contains a mixture of strains of the Sars-CoV-2 virus (original variant (Wuhan strain) and Ômicron variant). Its use was authorized for administration as a single booster dose, that is, it can only be applied to those who have already been vaccinated against the disease (with one or two doses, depending on the vaccine). Authorized for adult and pediatric use from 6 years of age. Produced from synthetic messenger RNA [11].

**Bivalent Comirnaty BA.4/BA.5 (Pfizer):** registration granted on 07/24/2023

Vaccine indicated as a booster dose and should be applied starting three months after the primary vaccine series or previous booster. Recommended for the population from 6 months of age and produced from synthetic messenger RNA [12].

**Covid-19 vaccine (recombinant) (Zalika):** registration granted on 01/08/2024

Updated vaccine for the XBB.1.5 omicron strain. Recommended for individuals over 12 years of age. The primary vaccination schedule consists of 2 doses, administered 21 days apart. A booster dose of the vaccine is recommended at least 2 months after immunization with any vaccine against Covid-19. Produced from recombinant S (spike) protein + adjuvant [13].

**Spikevax (Adium):** registration granted on 03/06/2024

Updated vaccine for the ômicron XBB.1.5 strain, authorized for use in individuals from 6 months of age. Produced from synthetic messenger RNA [14].

**References:**

1. Brasil. Ministério da Saúde. Agência Nacional de Vigilância Sanitária - Anvisa. Coronavac (Butantan) [Internet]. Available from: https://www.gov.br/anvisa/pt-br/assuntos/paf/coronavirus/vacinas/coronavac. Accessed in: May 14, 2024.

2. Brasil. Ministério da Saúde. Agência Nacional de Vigilância Sanitária - Anvisa. Comirnaty bivalente B.A 1 (Pfizer) [Internet]. Available from: https://www.gov.br/anvisa/pt-br/assuntos/paf/coronavirus/vacinas/comirnaty-bivalente-b-a-1-pfizer. Accessed in: May 14, 2024.

3. Brasil. Ministério da Saúde. Agência Nacional de Vigilância Sanitária - Anvisa. Sputnik [Internet]. Available from: https://www.gov.br/anvisa/pt-br/assuntos/paf/coronavirus/vacinas/sputnik. Accessed in: May 14, 2024.

4. Nishioka SA. O que aconteceu com a vacina russa, Sputnik V, da qual há muito não se ouve falar por aqui? [Internet]. Universidade Nacional Aberta do SUS - UNA-SUS. Available from: https://www.unasus.gov.br/especial/covid19/markdown/648. Accessed in: May 14, 2024.

5. Herrera‑Comoglio R, Lane S. Vaccine-induced immune thrombocytopenia and thrombosis after the Sputnik V vaccine. N Engl J Med 2022; 387:1431-1432. https://doi.org/10.1056/NEJMc2210813.

6. Brasil. Ministério da Saúde. Agência Nacional de Vigilância Sanitária - Anvisa. Covaxin (SUSPENSA) [Internet]. Available from: https://www.gov.br/anvisa/pt-br/assuntos/paf/coronavirus/vacinas/covaxin. Accessed in: May 14, 2024.

7. Brasil. Ministério da Saúde. Agência Nacional de Vigilância Sanitária - Anvisa. Anvisa suspende de forma cautelar estudo da Covaxin no Brasil (2021) [Internet]. Available from: https://www.gov.br/anvisa/pt-br/assuntos/noticias-anvisa/2021/anvisa-suspende-de-forma-cautelar-estudo-da-covaxin-no-brasil. Accessed in: May 14, 2024.

8. Brasil. Ministério da Saúde. Agência Nacional de Vigilância Sanitária - Anvisa. Comirnaty (Pfizer/Wyeth) [Internet]. Available from: https://www.gov.br/anvisa/pt-br/assuntos/paf/coronavirus/vacinas/pfizer. Accessed in: May 14, 2024.

9. Brasil. Ministério da Saúde. Agência Nacional de Vigilância Sanitária - Anvisa. Oxford/Covishield (Fiocruz e Astrazeneca) [Internet]. Available from: https://www.gov.br/anvisa/pt-br/assuntos/paf/coronavirus/vacinas/astrazeneca. Accessed in: May 14, 2024.

10. Brasil. Ministério da Saúde. Agência Nacional de Vigilância Sanitária - Anvisa. Janssen Vaccine (Janssen-Cilag) [Internet]. Available from: https://www.gov.br/anvisa/pt-br/assuntos/paf/coronavirus/vacinas/janssen. Accessed in: May 14, 2024.

11. Brasil. Ministério da Saúde. Agência Nacional de Vigilância Sanitária - Anvisa. Spikevax bivalente (Adium) [Internet]. Available from: https://www.gov.br/anvisa/pt-br/assuntos/paf/coronavirus/vacinas/spikevax-bivalente. Accessed in: May 14, 2024.

12. Brasil. Ministério da Saúde. Agência Nacional de Vigilância Sanitária - Anvisa. Comirnaty bivalente BA.4/BA.5 (Pfizer) [Internet]. Available from: https://www.gov.br/anvisa/pt-br/assuntos/paf/coronavirus/vacinas/comirnaty-bivalente-pfizer. Accessed in: May 14, 2024.

13. Brasil. Ministério da Saúde. Agência Nacional de Vigilância Sanitária - Anvisa. Vacina Covid-19 (recombinante) (Zalika) [Internet]. Available from: https://www.gov.br/anvisa/pt-br/assuntos/paf/coronavirus/vacinas/zalika. Accessed in: May 14, 2024.

14. Brasil. Ministério da Saúde. Agência Nacional de Vigilância Sanitária - Anvisa. Spikevax bivalente (Adium) [Internet]. Available from: https://www.gov.br/anvisa/pt-br/assuntos/paf/coronavirus/vacinas/spikevax-bivalente. Accessed in: May 14, 2024.
